# Supplementary material for: Structural insights into mechanisms of zinc scavenging by the Candida albicans zincophore Pra1
Source: Nat Commun. 2025 Nov 28;16:10753. doi: 10.1038/s41467-025-65782-0 (PMC12663325; doi:10.1038/s41467-025-65782-0)
Supplement: Supplementary file 1 — Supplementary Information [file 41467_2025_65782_MOESM1_ESM.pdf]

Title: Structural insights into mechanisms of zinc scavenging by the *Candida albicans* zincophore Pra1

Authors: Alexandre Nore, Elena Roselletti, Tanmoy Chakraborty, Nicha Särkkä, Rajika L Perera, Duncan Wilson* and Johanna Syrjänen*

Correspondence to: johanna.syrjanen@helsinki.fi and duncan.wilson@exeter.ac.uk

Supplementary Figures and Figure Legends

Supplementary Figure 1. Size exclusion chromatography (SEC) and SDS-PAGE of recombinantly expressed and purified *Candida albicans* Pra1. a) A SEC profile of wild type Pra1 analysed using a Superose 6 10/300 Increase column in 20 mM HEPES-NaOH pH 7.4, 300 mM NaCl, 0.5 mM EDTA buffer. **b)** SDS-PAGE gel of the TEV-cleaved peak Pra1 fractions. The smeariness of the bands in the SDS-PAGE gel is consistent with extensive glycosylation of the Pra1 protein. The ladder indicates molecular weights in kDa.

Supplementary Figure 2. Workflow of cryo-EM structure determination of *C.albicans* Pra1 in the presence of zinc. a) A representative micrograph. **b)** Representative 2D classes. **c)** Overview of the data processing pipeline. Initial motion correction, CTF estimation and particle picking were performed in Warp v.1.0.9, other steps were performed in cryoSPARC v.4.3.1 [1,2]. In cryoSPARC v.4.3.1, a subset of particles was used for 2D classification. Selected particles were then used for *ab initio* volume generation. These volumes were used as inputs for the first round of heterogenous refinement using all 3,486,720 particles (6 classes). Following two more rounds of heterogenous refinement (with 4 and 5 classes, respectively), homogenous refinement and CTF refinement were performed. These data were then used to generated two maps with different imposed symmetries: c1 (no symmetry) and c3 using non-uniform refinement. The c1 map was used for subsequent analyses and deposited. **d)** The Fourier Shell Correlation graph of the final c1 map. **e)** Model built into the cryo-EM map. **f)** A local resolution map of the final c1 map calculated using ResMap v.1.1.4 [3]. **g)** The viewing direction distribution plot showing the diversity of particle orientations in the dataset.

Supplementary Figure 3. The cryo-EM map of *C. albicans* Pra1 exhibits extensive N-linked glycosylation. The unsharpened cryo-EM map shown here was determined without imposing symmetry (c1). From the cryo-EM density map and consequent model building, we observe that residues N48, N89, N135 and N208 are glycosylated. The glycosylation was not resolved at sufficient resolution to unambiguously build specific oligosaccharides. These glycans are indicated in grey. **a)** Two glycans (shown here as an N48 residue from subunit E in smudge green and an N48 residue from subunit D in cornflower blue) are in close proximity to the inter-dimer interface. The inter-dimer interface is indicated by a rectangle outlined in a dashed black line. **b)** Six glycans (Shown here are N89, N208, N135 from subunit D and N89, N208, N135 from subunit C) are in close proximity to the intra-dimer interface. The intra-dimer interface is indicated by a rectangle outlined in a dashed black line. Subunit C is in sky blue and subunit D is in cornflower blue.

Supplementary Figure 4. *C. albicans* Pra1 superposed with the metalloprotease deuterolysin. a) The overall fold of a Pra1 subunit is similar to that of deuterolysin (PDB code: 1EB6) as indicated by a structural superposition of the two proteins in PyMOL 2.5.4. **b)** The canonical catalytically active residues in HEXXH+D type metalloproteases correspond to residues E143 and D129 in deuterolysin [4]. E143 is required for deuterolysin catalytic activity and is not conserved in *C. albicans* Pra1. In Pra1, H193 is in this position. Furthermore, Deuterolysin D129 is also crucial for catalytic activity and is located in the same position as Pra1 R179. However, the Pra1 R179 sidechain does not point towards the zinc coordination site like D129 points toward the catalytic site. Based on this structural overlay, it appears that the catalytic site of deuterolysin corresponds to a Zn^{2+} binding site in Pra1. For clarity, only the zinc from PBD-1EB6 is shown. Pra1 is shown in green and deuterolysin is shown in grey.

Supplementary Figure 5. The distances of the histidine residues from Zn^{2+} in the histidine triad vary by subunit. The N3-nitrogen (indicated in panel **a**) of the imidazole ring in His 178, His 182 and His 193 participate in Zn^{2+} coordination. For completeness, the position of the N1 nitrogen is also indicated. Panel a shows that the distance from the centre of the Zn^{2+} ion to the centre of the N3-nitrogen in His 193 and His 182 is 2.3 Å in subunit A. In contrast, the distance from the centre of the Zn^{2+} ion to the centre of the N3-nitrogen in His 178 is 2.7 Å. The distances are indicated by the dashed lines. Panels **b-f** show the histidine

triads with a Zn^{2+} ion in the other subunits. In most other subunits, the Zn^{2+} ion is equidistant from the histidines in the triad, at a distance of approximately 2.3 Å. The cryo-EM density map is visualised at the same contour level in each panel.

Supplementary Figure 6. Workflow of cryo-EM structure determination of *C.albicans* Pra1 in the absence of zinc. a) A representative micrograph. **b)** Representative 2D classes. **c)** Overview of the data processing pipeline. Initial motion correction, CTF estimation and particle picking were performed in Warp v.1.0.9, other steps were performed in cryoSPARC v.4.4.0 [1,2]. A subset of particles were used for 2D classification in cryoSPARC v.4.4.0. Selected “junk” and good particles were used for *ab initio* volume generation. These volumes were used as inputs for the first round of heterogenous refinement using all 1,025,587 particles (5 classes). A second round of heterogenous refinement (5 classes) was performed to further remove “junk” particles from the dataset.The cleaned-up data were then used to generated two maps with different imposed symmetries: c1 (no symmetry) and c3 using non-uniform refinement. The c1 map was used for subsequent analyses and deposited. **d)** The Fourier Shell Correlation graph of the c1 map. **e)** Model built into the cryo-EM map. **f)** A local resolution map of the final c1 map calculated using ResMap v.1.1.4[3]. **g)** The viewing direction distribution plot showing the diversity of particle orientations in the dataset.

Supplementary Figure 7. A structural superposition of the Pra1 hexamers in the presence and absence of Zn^{2+} shows that Zn^{2+} binding does not induce global changes to the Pra1 protein structure. The models of the final c1 maps corresponding to Pra1 in the presence and absence of Zn^{2+} were superposed in Pymol 2.5.4. The RMSD of 0.175 Å indicates that Zn^{2+} binding does not induce global changes to the resolved parts of the structure. Pra1 in the absence of Zn^{2+} is shown in purple and Pra1 in complex with Zn^{2+} is shown in green.

Supplementary Figure 8. AlphaFold predicted structure of a Pra1 monomer. Using a confidence score from 1-100, the core of the protein is predicted with very high confidence (>90; dark blue), the C-terminal motif with high confidence (>70; aqua) and the unstructured region with low (>50) to very low confidence (<50) [5,6].

Supplementary Figure 9. Sequence alignment of Deuterolysin from *Aspergillus oryzae* and Pra1 orthologues basidiomycetes (*Ustilago maydis*, *Cryptococcus depauperatus*), chytrids (*Spizellomyces punctatus*) and ascomycetes (*Blastomyces percusus*, *Candida albicans* and *Aspergillus fumigatus*). Calculated using ESPript - https://espript.ibcp.fr [7].

Supplementary Figure 10. Size exclusion chromatography (SEC) of recombinantly expressed and pu rified
***Candida albicans* Pra1 wild type protein and H178A/H182A mutant Pra1 protein.**The proteins were analysed using a Superose 6 10/300 Increase column in 20 mM HEPES-NaOH pH 7.4, 300 mM NaCl, 0.5 mM EDTA buffer. An overlay of the SEC profiles shows that the peak retention volume of both wild type Pra1 and H178A/H182A mutant Pra1 is coincident at 14.2 ml, as indicated by the dashed grey line. The identical retention peak volume and the peak shape demonstrate that the wild type Pra1 protein and the H178A/H182A mutant protein, although functionally different, form similar structural assemblies.

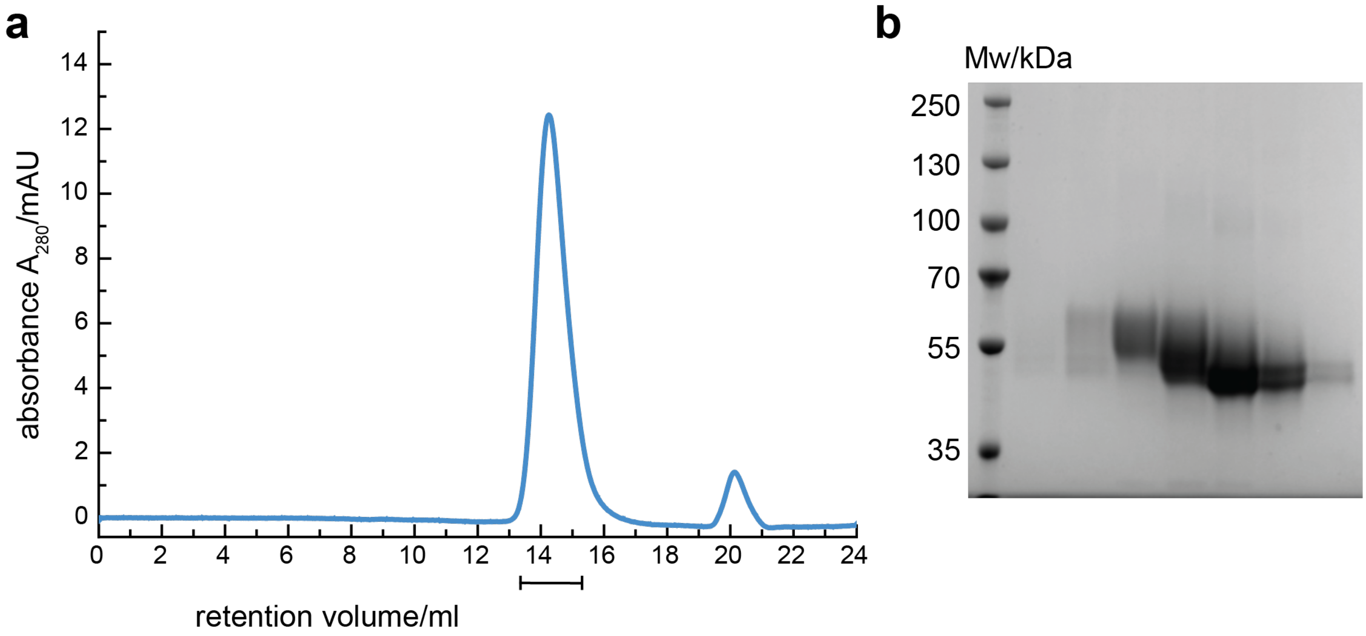

Supplementary Figure 1

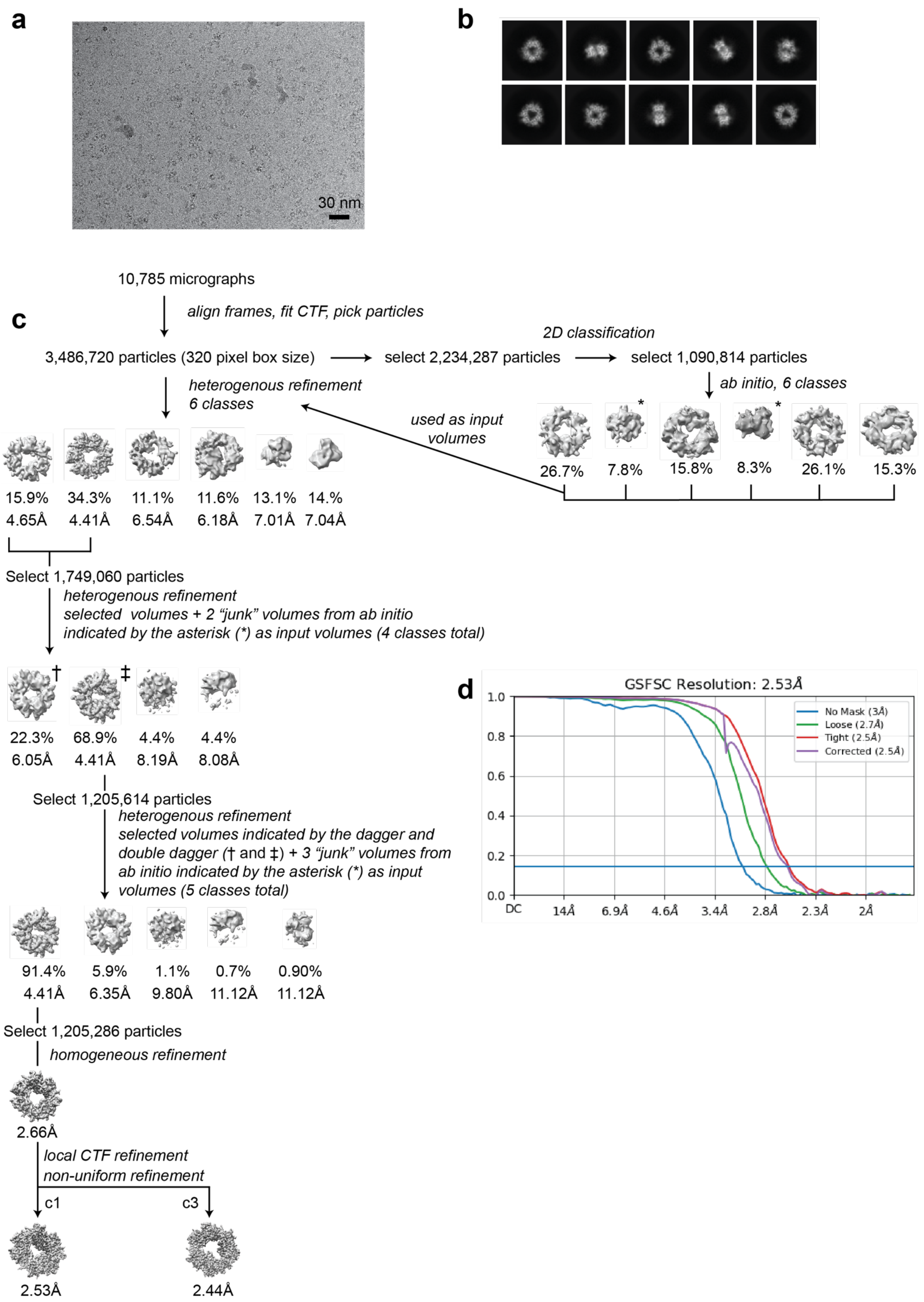
a
30 nm
b
c
10,785 micrographs
align frames, fit CTF, pick particles
3,486,720 particles (320 pixel box size)
select 2,234,287 particles
2D classification
select 1,090,814 particles
ab initio, 6 classes
26.7%
7.8%
15.8%
8.3%
26.1%
15.3%
used as input volumes
heterogenous refinement
6 classes
15.9% 4.65Å
34.3% 4.41Å
11.1% 6.54Å
11.6% 6.18Å
13.1% 7.01Å
14.% 7.04Å
Select 1,749,060 particles
heterogenous refinement
selected volumes + 2 “junk” volumes from ab initio
indicated by the asterisk (*) as input volumes (4 classes total)
22.3% 6.05Å
68.9% 4.41Å
4.4% 8.19Å
4.4% 8.08Å
Select 1,205,614 particles
heterogenous refinement
selected volumes indicated by the dagger and
double dagger († and ‡) + 3 “junk” volumes from
ab initio indicated by the asterisk (*) as input
volumes (5 classes total)
91.4% 4.41Å
5.9% 6.35Å
1.1% 9.80Å
0.7% 11.12Å
0.90% 11.12Å
Select 1,205,286 particles
homogeneous refinement
2.66Å
local CTF refinement
non-uniform refinement
c1
c3
2.53Å
2.44Å
d
GSFSC Resolution: 2.53Å
No Mask (3Å)
Loose (2.7Å)
Tight (2.5Å)
Corrected (2.5Å)
1.0
0.8
0.6
0.4
0.2
0.0
DC
14Å
6.9Å
4.6Å
3.4Å
2.8Å
2.3Å
2Å

e

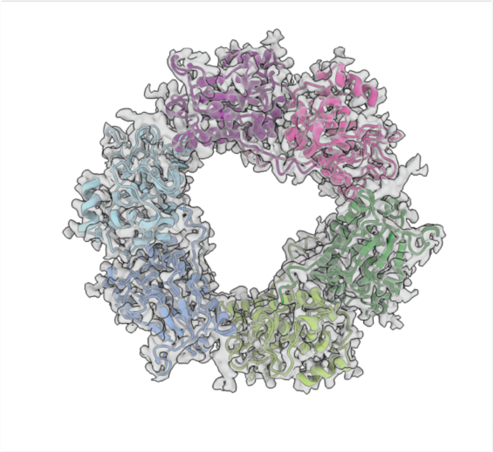

f

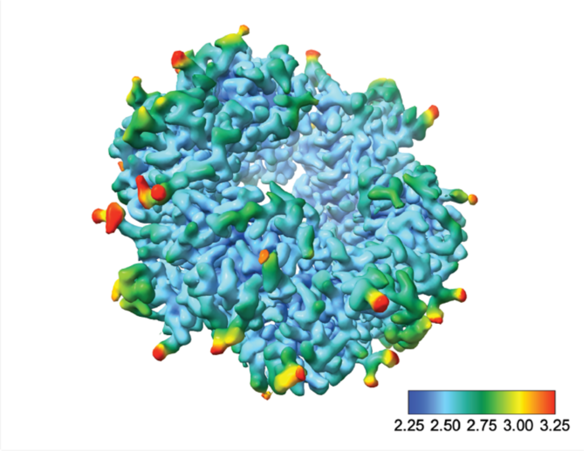

g

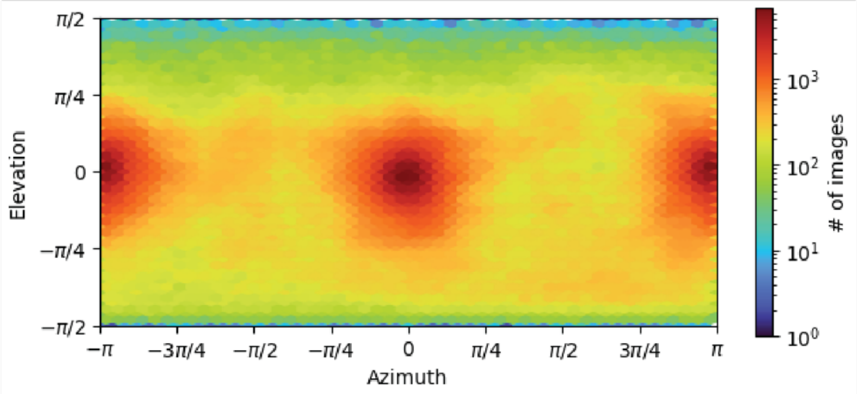

Supplementary Figure 2

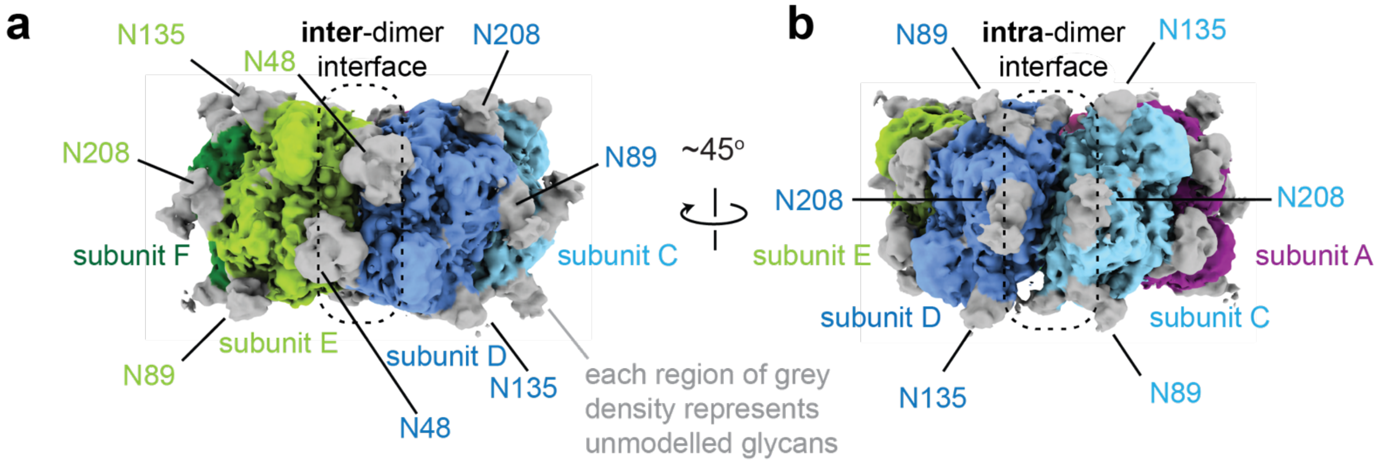

Supplementary Figure 3

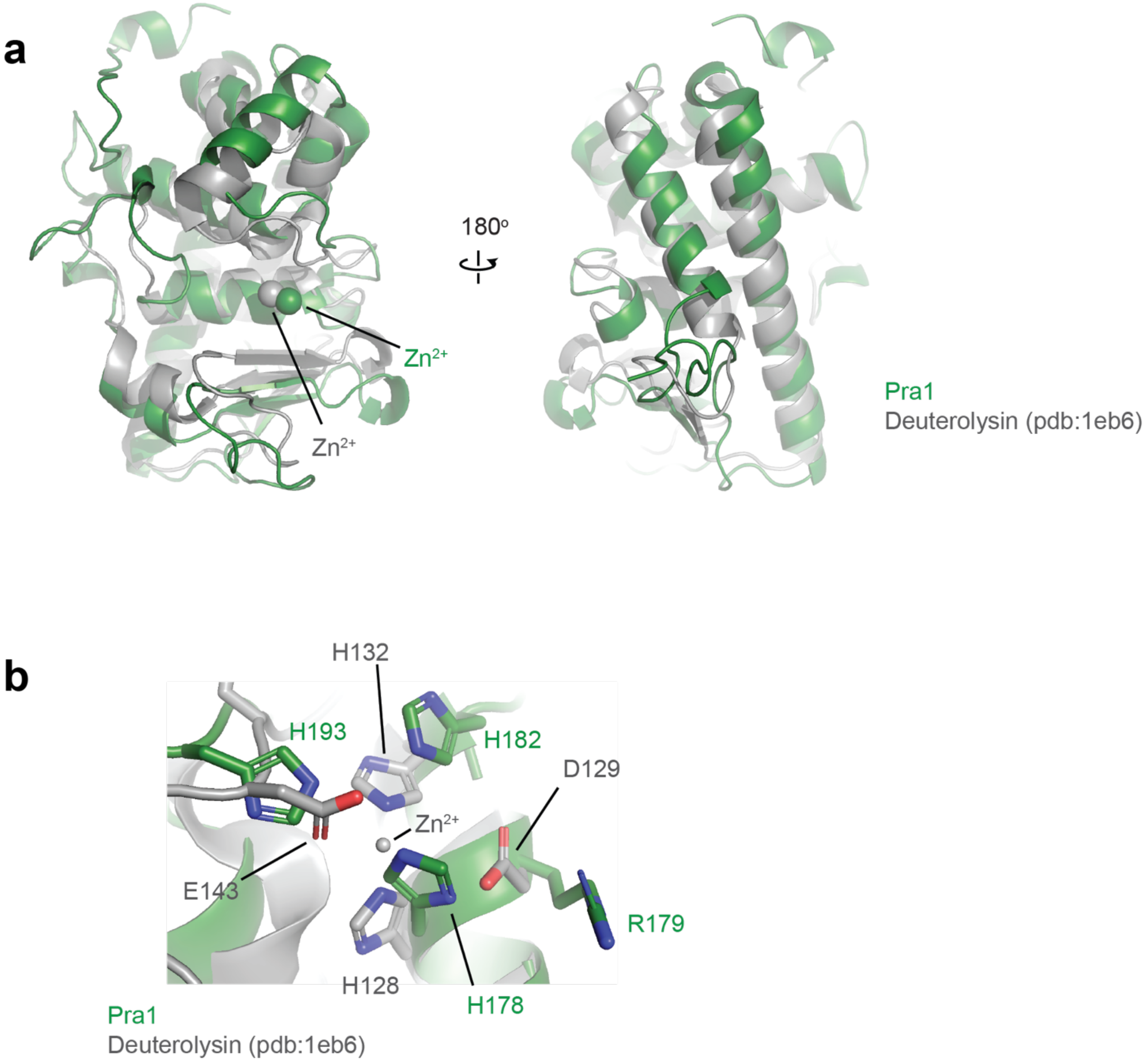

Supplementary Figure 4

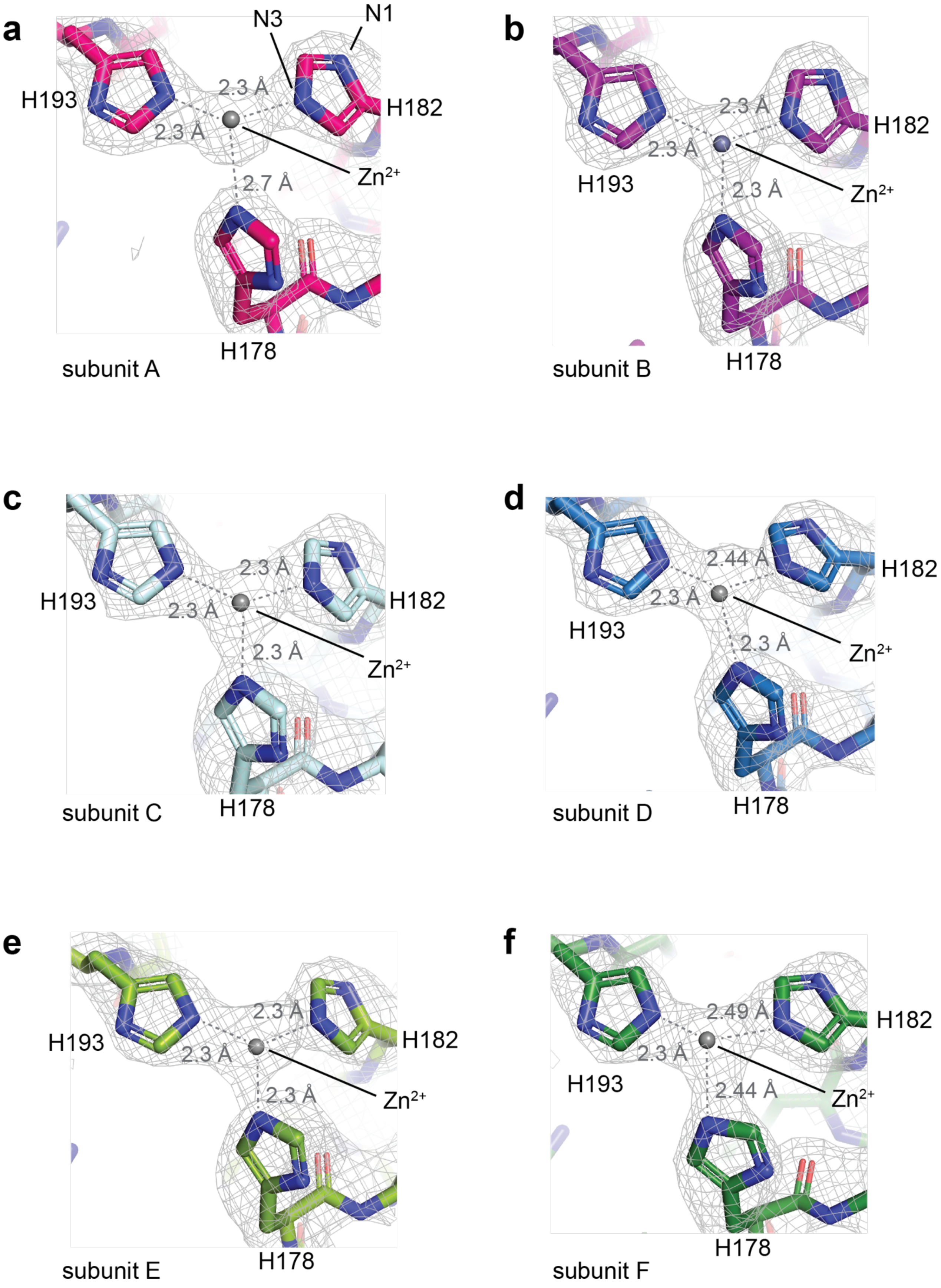

Supplementary Figure 5

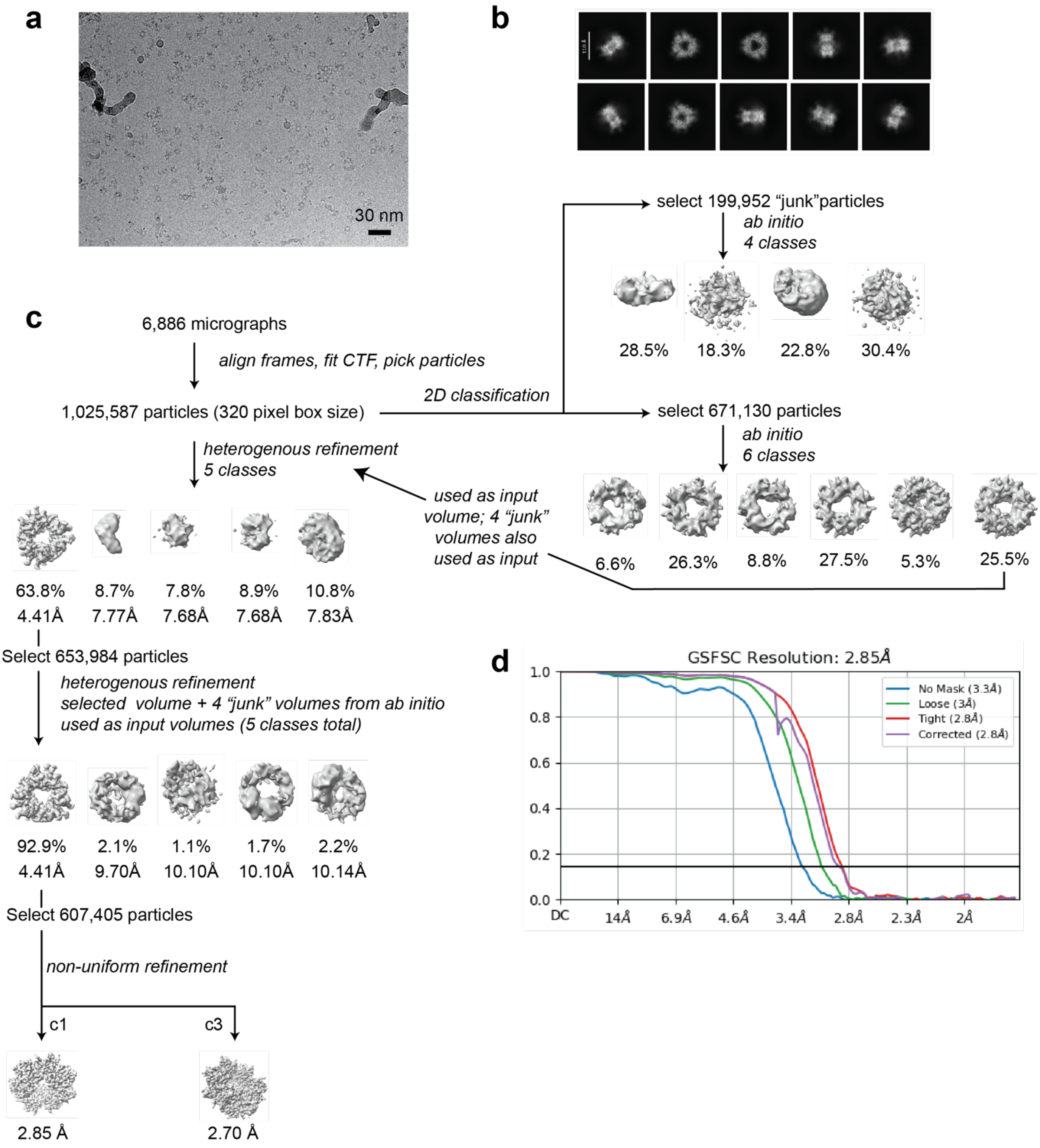

a
30 nm
b
c
6,886 micrographs
align frames, fit CTF, pick particles
1,025,587 particles (320 pixel box size)
2D classification
select 199,952 "junk"particles
ab initio
4 classes
28.5%
18.3%
22.8%
30.4%
select 671,130 particles
ab initio
6 classes
6.6%
26.3%
8.8%
27.5%
5.3%
25.5%
used as input volume; 4 "junk" volumes also used as input
heterogenous refinement
5 classes
63.8%
8.7%
7.8%
8.9%
10.8%
4.41Å
7.77Å
7.68Å
7.68Å
7.83Å
Select 653,984 particles
heterogenous refinement
selected volume + 4 "junk" volumes from ab initio used as input volumes (5 classes total)
92.9%
2.1%
1.1%
1.7%
2.2%
4.41Å
9.70Å
10.10Å
10.10Å
10.14Å
Select 607,405 particles
non-uniform refinement
c1
c3
2.85 Å
2.70 Å
d
GSFSC Resolution: 2.85Å
No Mask (3.3Å)
Loose (3Å)
Tight (2.8Å)
Corrected (2.8Å)
1.0
0.8
0.6
0.4
0.2
0.0
DC
14Å
6.9Å
4.6Å
3.4Å
2.8Å
2.3Å
2Å

e

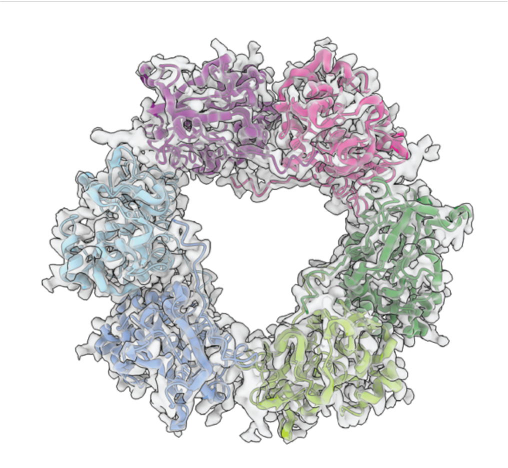

f

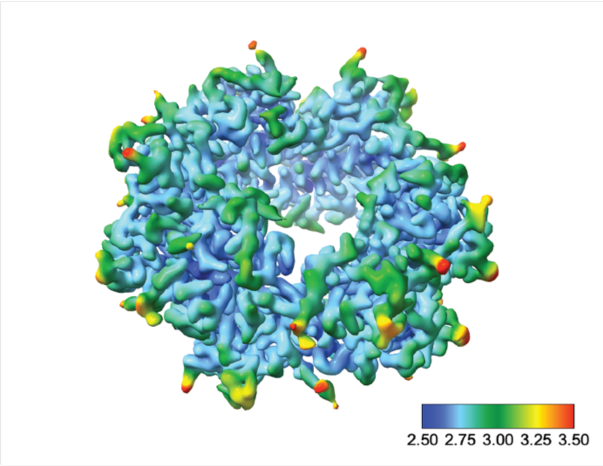

g

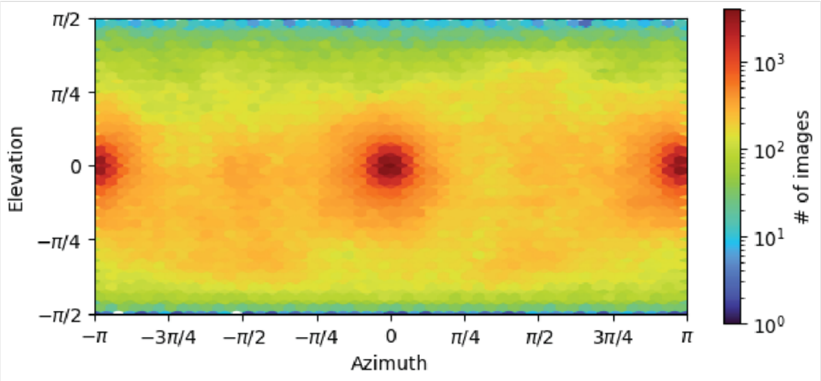

Supplementary Figure 6

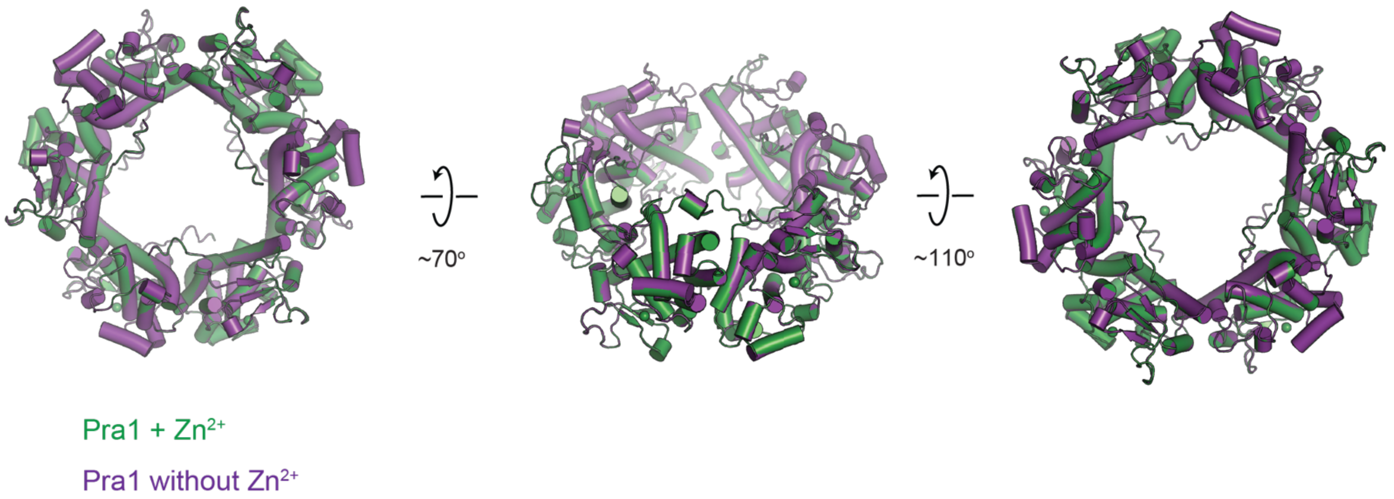

Supplementary Figure 7

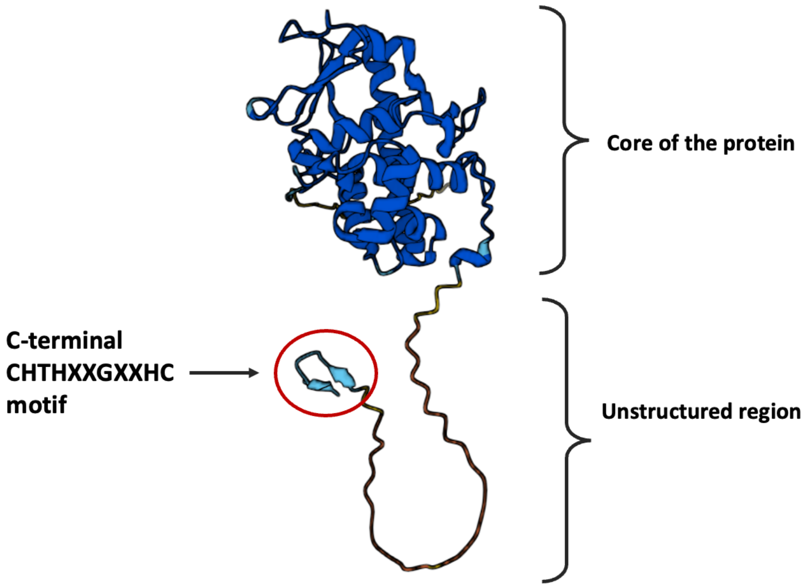

Supplementary Figure 8

```
Deuterolysin                     VTKALSQLTRRTEVTDCKGDAESSLTTALSNAAKLANQAAEAA--ESGDESKFEEYFKTT   222
Ustilago_maydis                  -----QIDSVWRIHESCNGTQRAQISSGIDDMKKLAHN-SINHILNYPKDEFFIKYFGQD   93
Cryptococcus_depauperatus        ------YTSDINIHESCNATQRRMLDKALSDAFEVASFAKEYARTNGPADPVFQKYFGKD   80
Spizellomyces_punctatus          -RRQLPPSTRVQMHSSCSASQKQTLNKALDDMNKLTIHATKRILDKTYEDPVYQTYFGNG   81
Rhizophlyctis_rosea              LSNGKGYNADVTIHASCKGTQERALTKALGEMNDLAKIAANRILKYGSEDELYKKYFGDG   91
Blastomyces_percursus            RKCGYGMMDPYPIHDSCNATERRMISRGLDDAITLAAHARDHVLKFGHDSSLYRKYFGNA   133
Candida_albicans_Pra1            ------WVKGFPIDSSCNATQYNQLSTGLQEAQLLAEHARDHTLRFGSKSPFFRKYFGNE   90
Aspergillus_fumigatus_AspF2      ------AVTSFPIHSSCNATQRRQIEAGLNEAVELARHAKAHILRWGNESEIYRKYFGNR   103
                                                .*..     :  .: :   ::             .  :  **

Deuterolysin                     DQQTRTTVAERLRAVAKEAGSTSGGSTTYHCNDPYGYCEP-NVLAYT---LPSKNEIANC   278
Ustilago_maydis                  ADPA-PVVGYYVEL-----VYGNKGDALLRCDNPDDNCRL-PEWNGHWRGNNATAETVIC   146
Cryptococcus_depauperatus        ADSYTQVIGIWDAF-----LTGNKEGVILRCDNPDGNCAQ-KGFNGHWRGNNATSETVIC   134
Spizellomyces_punctatus          ES-A-TVVGYYGIL-----TAGK-----VFFSDVDNKCSQ-PGWAGHWRGEVAPLETVIC   128
Rhizophlyctis_rosea              EA-A-TVLGYYKTI-----LYGNKPGVLFRCDNIDNKCHQ-EGWAGHWRTEIAPLETVIC   143
Blastomyces_percursus            PT-S-NVIGNLARI-----VDGNRKKTLFRCDDPDGNCKRIPTYGGHWRGENATDETVIC   186
Candida_albicans_Pra1            TASA-EVVGHFDNV-----VGADKSSILFLCDDLDDKCKN-DGWAGYWRGSNHSDQTIIC   143
Aspergillus_fumigatus_AspF2      PT-M-EAVGAYDVI-----VNGDKANVLFRCDNPDGNCAL-EGWGGHWRGANATSETVIC   155
                                       .:.             .        .:  . *                 :   *

Deuterolysin                     DIYYS--ELPPLAQKCHAQDQA----------TTTLHEFTHAPGVYQ-PGTEDLGYGYDA   325
Ustilago_maydis                  ELSYV--TRRPLEKLCSAGFQLGTDNPSLYFGADLMHRAFHVPEFVH-EKIHHYADSYAD   203
Cryptococcus_depauperatus        DLSYT--SRIYNEAFCMSGFQLASQKPSTYWSVDLIHRFFHVPAVTN-GLVGHFAEDYAS   191
Spizellomyces_punctatus          PLSFNTTARKPLSALCQDGDRFSQHKTNYFLSSDLMHRLFHVPLINPEERVDHYASNYTE   188
Rhizophlyctis_rosea              PLSFT-DARKPLSAICKNGNKISAVKSNYFFASDLMHRLFHVPLINPGERVDHFAGNWTE   202
Blastomyces_percursus            ELSYK--TRLYLEHFCMGGYTVAKSPRNTYFGLDMMHRLYHMPAIGE-NHVGHFADTYND   243
Candida_albicans_Pra1            DLSFV--TRRYLTQLCSSGYTVSKSKTNIFWAGDLLHRFWHLKSIGQ-LVIEHYADTYEE   200
Aspergillus_fumigatus_AspF2      DRSYT--TRRWLVSMCSQGYTVAGSETNTFWASDLMHRLYHVPAVGQ-GWVDHFADGYDE   212
                                    :           *                   :*.  *   .       . .  :

Deuterolysin                     ------------------------------------------------------------   352
Ustilago_maydis                  APTST--------PAA---------------------------------TPTPSAASSD   278
Cryptococcus_depauperatus        ATSSNA----GAASASVPPPPAESTGCTLHGDHYHCTGPATPTTQAAEVHDTPASGGDKD   307
Spizellomyces_punctatus          ------------------------------------------------------------   227
Rhizophlyctis_rosea              ------------------------------------------------------------   241
Blastomyces_percursus            ATQ-PT-------KG-TPTVP---------------------------HPPTPTADVPRV   321
Candida_albicans_Pra1            -SG-SDSGASSTASSSHQHTD---------------------------SNPSATTDANSH   288
Aspergillus_fumigatus_AspF2      ASTSTSSSSSGSGSGATTTPT---------------------------DSPSATIDVPPN   302


Deuterolysin                     -------------- 352
Ustilago_maydis                  CHTHADGSIHCGTH 292
Cryptococcus_depauperatus        CHTHADGTVHCV-- 319
Spizellomyces_punctatus          -------------- 227
Rhizophlyctis_rosea              -------------- 241
Blastomyces_percursus            G------------- 322
Candida_albicans_Pra1            CHTHADGEVHC--- 299
Aspergillus_fumigatus_AspF2      CHTHEGGQLHCT-- 314
```

Supplementary Figure 9

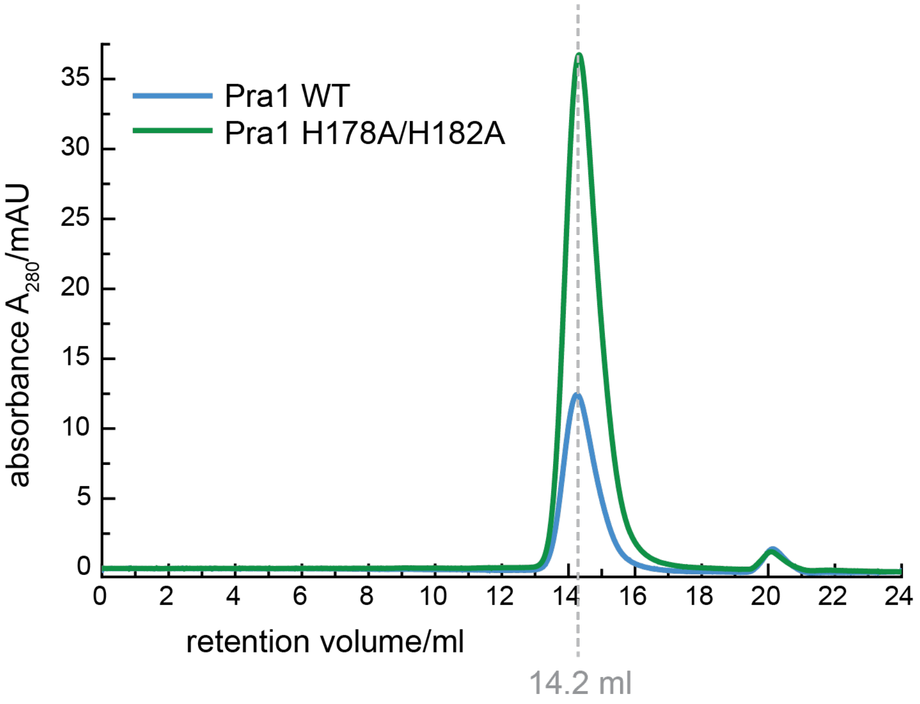

Supplementary Figure 10

References

1. Tegunov, D. & Cramer, P. Real-time cryo-electron microscopy data preprocessing with Warp. *Nat. Methods* **16**, 1146–1152 (2019).

2. Punjani, A., Rubinstein, J. L., Fleet, D. J. & Brubaker, M. A. cryoSPARC: algorithms for rapid unsupervised cryo-EM structure determination. *Nat. Methods* **14**, 290–296 (2017).

3. Kucukelbir, A., Sigworth, F. J. & Tagare, H. D. Quantifying the local resolution of cryo-EM density maps. *Nat. Methods* **11**, 63–65 (2014).

4. Fushimi, N., Ee, C. E., Nakajima, T. & Ichishima, E. Aspzincin, a Family of Metalloendopeptidases with a New Zinc-binding Motif. *J. Biol. Chem.* **274**, 24195–24201 (1999).

5. Jumper, J. *et al.* Highly accurate protein structure prediction with AlphaFold. *Nature* **596**, 583–589 (2021).

6. Steinegger, M. & Söding, J. Clustering huge protein sequence sets in linear time. *Nat. Commun.* **9**, 2542 (2018).

7. Robert, X. & Gouet, P. Deciphering key features in protein structures with the new ENDscript server. *Nucleic Acids Res.* **42**, W320–W324 (2014).
