# Supplementary material for: Structural insights into mechanisms of zinc scavenging by the Candida albicans zincophore Pra1
Source: Nat Commun. 2025 Nov 28;16:10753. doi: 10.1038/s41467-025-65782-0 (PMC12663325; doi:10.1038/s41467-025-65782-0)
Supplement: Supplementary file 2 — Reporting Summary [file 41467_2025_65782_MOESM2_ESM.pdf]

nature portfolio

Corresponding author(s): Johanna Syrjanen and Duncan Wilson

Last updated by author(s): 13/10/25

Reporting Summary

Nature Portfolio wishes to improve the reproducibility of the work that we publish. This form provides structure for consistency and transparency in reporting. For further information on Nature Portfolio policies, see our Editorial Policies and the Editorial Policy Checklist.

Statistics

For all statistical analyses, confirm that the following items are present in the figure legend, table legend, main text, or Methods section.

n/a	Confirmed	
☐	☒	The exact sample size (*n*) for each experimental group/condition, given as a discrete number and unit of measurement
☒	☐	A statement on whether measurements were taken from distinct samples or whether the same sample was measured repeatedly
☐	☒	The statistical test(s) used AND whether they are one- or two-sided<br>*Only common tests should be described solely by name; describe more complex techniques in the Methods section.*
☒	☐	A description of all covariates tested
☐	☒	A description of any assumptions or corrections, such as tests of normality and adjustment for multiple comparisons
☐	☒	A full description of the statistical parameters including central tendency (e.g. means) or other basic estimates (e.g. regression coefficient) AND variation (e.g. standard deviation) or associated estimates of uncertainty (e.g. confidence intervals)
☐	☒	For null hypothesis testing, the test statistic (e.g. *F*, *t*, *r*) with confidence intervals, effect sizes, degrees of freedom and *P* value noted<br>*Give P values as exact values whenever suitable.*
☒	☐	For Bayesian analysis, information on the choice of priors and Markov chain Monte Carlo settings
☒	☐	For hierarchical and complex designs, identification of the appropriate level for tests and full reporting of outcomes
☒	☐	Estimates of effect sizes (e.g. Cohen's *d*, Pearson's *r*), indicating how they were calculated

Our web collection on statistics for biologists contains articles on many of the points above.

Software and code

Policy information about availability of computer code

Data collection	EPU 2.10.0.5 was used for cryo-EM data collection.
Data analysis	WARP 1.0.9 and CryoSPARC 4.4 were used for cryo-EM single particle analysis. Model building was performed in UCSF Chimera 1.16 and Coot 0.9.8.7. The models were refined against the cryo-EM maps in Coot 0.9.8.7 and PHENIX 1.21.2. Superpositions of atomic models and subsequent RMSD calculations were performed in Pymol 2.5.4 using the align algorithm.<br>Statistical analysis of Candida albicans growth curves (Figure 4) and neutrophil recognition (Figure 5) were performed in Graphpad Prism.

For manuscripts utilizing custom algorithms or software that are central to the research but not yet described in published literature, software must be made available to editors and reviewers. We strongly encourage code deposition in a community repository (e.g. GitHub). See the Nature Portfolio guidelines for submitting code & software for further information.

Data

Policy information about availability of data

All manuscripts must include a data availability statement. This statement should provide the following information, where applicable:

- Accession codes, unique identifiers, or web links for publicly available datasets
- A description of any restrictions on data availability
- For clinical datasets or third party data, please ensure that the statement adheres to our policy

The final cryo-EM maps have been deposited in the Electron Microscopy Data Bank under accession codes: EMD-48872 and EMD-48869. The final models have

been deposited in the Protein Data Bank under the accession codes: PDB-9N4D and PDB-9N47. All files with accession codes will be available on publication. The PDB entry 1EB6 was used for structural superposition analysis. The growth assay data and chemotaxis data (Figures 4 and 5) generated in this study are provided in the Source Data file.

Research involving human participants, their data, or biological material

Policy information about studies with human participants or human data. See also policy information about sex, gender (identity/presentation), and sexual orientation and race, ethnicity and racism.

Reporting on sex and gender	n/a
Reporting on race, ethnicity, or other socially relevant groupings	n/a
Population characteristics	n/a
Recruitment	n/a
Ethics oversight	n/a

Note that full information on the approval of the study protocol must also be provided in the manuscript.

Field-specific reporting

Please select the one below that is the best fit for your research. If you are not sure, read the appropriate sections before making your selection.

☒ Life sciences ☐ Behavioural & social sciences ☐ Ecological, evolutionary & environmental sciences

For a reference copy of the document with all sections, see nature.com/documents/nr-reporting-summary-flat.pdf

Life sciences study design

All studies must disclose on these points even when the disclosure is negative.

Sample size	Samples sizes for the Candida albicans growth curves (Figure 4) and neutrophil recognition assays (Figure 5) follows standard practice in the field. Candida albicans growth curves were replicated with n=8 per strain (Figure 4) and neutrophil recognition assays (Figure 5) were replicated with n=3 per strain. Cryo-EM sample size was determined by the availability of microscope time for data collection. The number of images is indicated in Supplementary Figures 2 and 6.
Data exclusions	Data were not excluded from growth assays or chemotaxis assays. Cryo-EM images with poor ice, high drift and poor CTF fits were excluded. Individual particles were excluded by 2D and 3D classification as outlined in Supplementary Figures 2 and 6, as is standard in the field.
Replication	Candida albicans growth curves were replicated with n=8 per strain (Figure 4) and neutrophil recognition assays (Figure 5) were replicated with n=3 per strain. Cryo-EM data were imaged from multiple grid squares and 1-2 grids, with the results being similar.
Randomization	Which yeast cells contributed to the growth measurements of a specific strain on a given day were random. Neutrophil recognition is an end-point assay and the total fluorescence of all migrated/recognized cells is measured at the end of the assay for each strain, so randomization is not applicable to this assay. Cryo-EM particles are randomised during data processing.
Blinding	Measuring the growth of different yeast strains or neutrophil recognition of different yeast strains does not depend on the researchers being blinded to these strains. Blinding is not technically or practically feasible for these cryo-EM studies as image analysis requires careful evaluation at each step of the data processing workflow. Researchers conducting data collection were also responsible for data analysis, excluding the possibility of blinding in these experiments.

Reporting for specific materials, systems and methods

We require information from authors about some types of materials, experimental systems and methods used in many studies. Here, indicate whether each material, system or method listed is relevant to your study. If you are not sure if a list item applies to your research, read the appropriate section before selecting a response.

Materials & experimental systems

n/a	Involved in the study
☒	☐ Antibodies
☐	☒ Eukaryotic cell lines
☒	☐ Palaeontology and archaeology
☐	☒ Animals and other organisms
☒	☐ Clinical data
☒	☐ Dual use research of concern
☒	☐ Plants

Methods

n/a	Involved in the study
☒	☐ ChIP-seq
☒	☐ Flow cytometry
☒	☐ MRI-based neuroimaging

Eukaryotic cell lines

Policy information about cell lines and Sex and Gender in Research

Cell line source(s)	Expi293F (Thermo Fisher) are derived from the 293 kidney (embryonic) cell line
Authentication	Authentication by Thermo Fisher
Mycoplasma contamination	Expi293F is declared to be mycoplasma-free by Thermo Fisher on arrival
Commonly misidentified lines (See ICLAC register)	n/a

Animals and other research organisms

Policy information about studies involving animals; ARRIVE guidelines recommended for reporting animal research, and Sex and Gender in Research

Laboratory animals	The study used Candida albicans fungal strains, which are listed in Table 2.
Wild animals	n/a
Reporting on sex	n/a
Field-collected samples	n/a
Ethics oversight	n/a

Note that full information on the approval of the study protocol must also be provided in the manuscript.

Plants

Seed stocks	n/a
Novel plant genotypes	n/a
Authentication	n/a
